# The RiVUR Study Outcomes and Implications on the Management of Vesicoureteral Reflux

**Published:** 2022

**Authors:** Tiffany Damm, Ranjiv Mathews

**Affiliations:** Southern Illinois University Medicine, Department of Surgery, Division of Urology, 747 N. Rutledge, 5th floor Springfield, IL 62702-9665, USA

**Keywords:** Vesicoureteral reflux, Antimicrobial prophylaxis, RiVUR trial, Reflux nephropathy, Ureteral reimplantation, Bladder and bowel dysfunction

## Abstract

The Randomized intervention for Vesicoureteral Reflux (RiVUR) study was an effort by the National Institute of Health to identify the most significant question on the management of vesicoureteral reflux (VUR), i.e. Did antibiotic prophylaxis reduce the incidence of recurrent urinary tract infections (UTI) in children with VUR? During the initial phases of the RiVUR study, several similar studies were performed that seemed to indicate a lack of benefit of antibiotic prophylaxis in VUR. However, few of these studies had the rigorous methodology and true randomization of the pediatric cohort that was studied in RiVUR. Additionally, many of these studies included children of wide age ranges and inconsistent assessments were used for identification of UTI and VUR. In 2011, the American Academy of Pediatrics (AAP) published a guideline statement for the evaluation of initial UTI in febrile children aged 2 to 24 months, which recommended against performing a Voiding Cystourethrogram (VCUG) in all children with a confirmed UTI.

The goal of the AAP guidelines was to reduce the number of VCUGs being performed and potentially to reduce the number of children diagnosed with low grade VUR that seems to have low potential to cause renal injury. The RiVUR study included over 600 children identified with VUR after a 1^st^ or 2^nd^ febrile UTI randomized to prophylaxis with trimethoprim/sulfamethoxazole (TMP/SMZ), or placebo and followed over a study timeline for 2 years. Overall, a 50% reduction was noted in the incidence of recurrent febrile UTI with the utilization of prophylaxis as compared to placebo. Additional sub-group analyses have been performed on the cohorts of the study; these are also evaluated in this review to determine the overall impact of the RiVUR study on the current management of VUR.

## Introduction

The National Institute of Health created the RiVUR study as a multi-institutional effort to evaluate the most important issues in the management of VUR. This effort included pediatricians, pediatric urologists, pediatric nephrologists, pediatric radiologists, and statisticians in a multidisciplinary effort to evaluate the role of antibiotic prophylaxis in the management of VUR [1,2]. In 2011 the AAP published a guideline statement for the management of initial UTI in febrile children aged 2 to 24 months that recommended against performing a VCUG after initial UTI [3]. This represented a shift from the long-standing evaluation of children presenting after a first febrile UTI, which included renal bladder ultrasound (RBUS) and voiding cystourethrogram (VCUG). Diagnosis of reflux was believed to be indicated, so that prophylaxis could be initiated to reduce the risk of recurrent UTIs and secondary renal scarring. This statement was published prior to the completion of RiVUR and was based on studies that had purported to evaluate the role of antimicrobial prophylaxis in VUR [4–9]. None of these studies employed the rigorous enrollment guidelines, age limitations, or other study parameters that underpinned RiVUR, making their data open to significant debate.

The standard management of vesicoureteral reflux has been controversial and the notion that VUR should be identified and treated to prevent renal scarring has been challenged [4,5]. Prior studies had suggested that recurrent infections in children with VUR were uncommon, especially in those with low grade VUR, and renal scarring was also noted to be uncommon [5,6]. The AAP guidelines therefore recommended that RBUS should be performed routinely for children with UTI and that VCUG was indicated only in those patients that had an abnormal RBUS [3]. RBUS has not been shown to be a good predictor for the presence of non-dilating VUR [10,11]. A logistical regression predictive model with RBUS findings for the detection of any VUR, was found to have a sensitivity of 86.3%, a specificity of 24.7% and a positive predictive value of 53.7% [10]. Sixty-two percent of children with a normal ultrasound after initial UTI in one study had grade 3 or higher VUR [11]. The rationale and results from the RiVUR trial are revisited in this review. In addition, a PubMed search was performed for any studies that utilized RiVUR participants for analysis.

## Outcomes and Implications

### Rationale and methods for the RiVUR trial

Randomized intervention for Vesicoureteral Reflux was a double-blind, randomized, placebo-controlled trial that enrolled for two years and randomized 609 participants aged 2 to 72 months of age who had a febrile UTI or symptomatic UTI and were concurrently diagnosed with VUR. The age range selected represented children who were most likely to have UTI in the context of VUR; in some studies, VUR was noted to be present in up to 30% of children presenting with UTI [12]. To capture a diverse population, participants were recruited from multiple centers and from a variety of clinical settings ranging from urology, nephrology, and pediatric clinics as well as the Emergency Department [13] Children were randomized to receive daily doses of either TMP/SMZ or placebo for 2 years. The RiVUR trial sought to determine if antibiotic prophylaxis in the setting of VUR had a significant impact on reduction of recurrent UTI and renal scarring, and to help identify those children who would benefit most from the non-surgical management of vesicoureteral reflux [14]. Other outcomes evaluated included renal scarring as assessed by dimercaptosuccinic acid (DMSA) renal scanning, renal function, development of antimicrobial resistance in fecal flora, compliance with prophylaxis and radiologic evaluation.

### Primary outcome from the RiVUR trial and implications

Most patients across both groups had VUR grade II and grade III. Compared to 24% of children who developed a recurrent UTI with placebo, 13% of children who received TMP/SMZ prophylaxis developed a recurrence of UTI [1]. The risk reduction was greatest in children that had an index infection that was febrile. This certainly suggests a benefit to the use of prophylaxis in the prevention of infection. Compliance remains a major concern with long term prophylaxis, and this was closely monitored in the context of the study. If compliance is poor, e.g. in routine practice, this benefit may be harder to achieve consistently. Therefore, when prophylactic regimens are instituted, counseling parents about the need for consistent administration of medications remains crucial for success.

The risk reduction for recurrence of UTI with use of antibiotic prophylaxis was also noted to be greater in specific subsets of children. In a reanalysis published in 2018 by Wang et al., there was a 3.7-fold increased risk for UTI in high-risk children that were not on antibiotic prophylaxis. High risk children included uncircumcised males with VUR Grade I-III, females with VUR grade I-III and bladder and bowel dysfunction (BBD), and all children with Grade IV VUR and BBD [15]. It is important to weigh the decision to initiate antibiotic prophylaxis in context of a child’s history and risk associated with recurrent UTI. Children who had an index infection that was febrile or had BBD at baseline had the greatest reduction of risk of UTI recurrence with the use of prophylaxis, with hazard ratio report at 0.41 and 0.21 respectively [1]. This lends additional credence to the need to aggressively manage bladder and bowel dysfunction in the context of UTI and VUR in children. Adverse events were similar in each group, with otitis media, diarrhea, pharyngitis, and rash being the most common [1]. These adverse events, however, were uncommon.

### Renal scarring and the RiVUR data

Although an effort was made to identify the role of prophylaxis in the prevention of renal scarring, several factors conspired to impact this parameter. Renal scarring was infrequently noted in this study cohort, with just 3.6% of renal scans indicating scarring. These rates were similar between the two groups (95.9% in the TMP/SMZ versus 96.9% in the placebo groups reported no renal scarring) [1]. Children with renal scarring tended to be older, had a second UTI before enrollment, and had high-grade VUR [2]. Additionally, worsening of scars was also noted infrequently because of 2-year timeline of the study.

Additional investigations using RiVUR data have been undertaken to examine the relationship of VUR and renal scarring. Although antibiotic prophylaxis was not directly reported to influence renal scarring in the RiVUR trial, secondary analysis showed that renal scarring was more likely with an increase in number of febrile UTIs. In a 2019 post hoc analysis by Shaikh et al., RiVUR and CUTIE (Careful Urinary tract evaluation Study) data were analyzed to determine the risk of renal scarring associated with the number of febrile UTIs. The CUTIE study was a sister study to RiVUR that enrolled patients that were not eligible for the RiVUR study. The incidence of renal scarring after 0, 1 or 2 febrile UTI was reported as 0%, 2.8% and 25.7%, respectively, and a second UTI conferred a 12-fold increase in risk for renal scarring [16].

In a secondary analysis of RiVUR participants who had follow up DMSA scans during and after the trial, recurrent UTI was found to be a risk factor for development of new renal scarring, and antibiotic prophylaxis decreased the rate of new renal scarring associated with recurrent UTI [17].

Delay in initiation of antimicrobial treatment for UTI lead to a higher rate of renal scarring; a delay of 48 hours increased odds of new renal scarring by approximately 48% [18]. Other predictors of renal scarring were interim UTIs (OR 6.44), Hispanic versus other (OR 5.24) and duration of fever prior to treatment (OR 1.008 for 1 hour increase) [19].

Although RiVUR data did not directly show a decrease in renal scarring with antibiotic prophylaxis in VUR, antibiotic prophylaxis should be considered to reduce the risk of renal scarring in children in higher risk cohorts. Parents of children at high-risk for renal scarring i.e. those with BBD, multiple UTIs, and vesicoureteral reflux should be counselled about the need to seek prompt evaluation and early treatment at the first sign of a febrile UTI.

## Additional Analysis of RiVUR Data

The large volume of data collected on this very well studied group of children has permitted additional variables and outcomes not part of the original intentions of the study to be reviewed.

### Radiographic studies associated with the diagnosis of VUR

In the RiVUR trial, VCUGs were read by a local clinical radiologist and two blinded reference radiologists. There was significant disagreement in grading even among trained local radiologists; only 59% of VUR grades were agreed upon by all three radiologists [20]. Grade of VUR is an important counseling parameter on the potential for spontaneous resolution, risk stratification and clinical decision-making in VUR treatment; this variability in grading therefore has a significant impact on clinical care and research trials. Besides presence and grade of VUR, other important information such as post void residual, shape of the bladder and delayed drainage could provide information about urinary retention, BBD, and Ureteropelvic junction obstruction that would be helpful in treating patients. Schaeffer et al. 2017 investigated VCUG reports performed in the RiVUR study and found that non-pediatric radiologists dictated a significantly less complete (6% fewer items) VCUG report; however, overall, reports omitted on average 50% of a checklist that was created to investigate completeness of reports [21]. A widely disseminated reporting template could help improve the completeness of VCUG reporting.

Radiographic studies used to diagnose VUR and identify renal scarring, VCUG and DMSA respectively, can be uncomfortable for children. VCUG requires that the child be catheterized, while a DMSA scan requires a child to remain immobile for 30–60 minutes after IV injection of DMSA, which is one reason the AAP guidelines sought to reduce VUR workup as a sweeping recommendation for all children with UTI. Shaikh et al. (2017) investigated the use of sedation on the discomfort of children undergoing DMSA scan. DMSA causes less discomfort than VCUG, and sedation appears to relieve discomfort associated with DMSA scan. Selective sedation of younger children (12–36 months) may be beneficial in reducing discomfort associated with DMSA scans [22]. Although radiographic imaging associated with VUR can be distressing, sedation should be utilized to preclude avoidance of imaging.

### BBD

The relationship of VUR with BBD was evaluated to determine the benefits of treatment of BBD in children with VUR [23]. Children must be evaluated for signs of BBD such as dribbling, urgency, incontinence, constipation, or behaviors that suppress bladder contractions (curtsy in girls, penis squeezing in boys), since these children have a higher prevalence of UTI. Reportedly, 54% of patients with BBD have UTIs versus 20% in the general population. The incidence of recurrent UTIs is increased in children who have both BBD and VUR (51%), which is higher than either BBD (35%) or VUR (20%) alone. In addition to a higher risk for recurrent UTI, concomitant BBD decreases the likelihood of spontaneous resolution of VUR [24]. Aggressive management of BBD with institution of voiding regimens, hydration and constipation control should therefore be an integral part of the management of potty-trained children with VUR.

### Antimicrobial resistance

An ongoing concern with the use of long-term prophylactic regimens is the potential for development of resistance. Although recurrent infections were usually resistant to TMP/SMZ, the overall incidence of resistant TMP/SMZ was only slightly higher in the antibiotic prophylaxis group as compared to placebo. TMP/SMZ resistance status of the index UTI was not associated with an increased risk of recurrent UTI in children treated with antibiotic prophylaxis [25]. Uncircumcised boys, children with BBD and Hispanic patients were more likely to experience antimicrobial resistance; these are also the same factors that account for increased risk of recurrence of UTI [23]. While concerning, in this randomized study the risk of resistant organisms remained insignificant.

### Cost-effectiveness

Palmer et al. evaluated the cost efficacy of the utilization of prophylaxis in patients with VUR. They utilized the outcomes of the RiVUR study, but incorporated costs of medications, imaging and the treatment of complications, such as pyelonephritis, likelihood for surgery and loss of parental work, based on Medicare reimbursement and data from the literature. Prophylaxis in this study was noted to be marginally more costly but led to significantly fewer infections. Even a small reduction in the cost of the antibiotic, would have led to an even greater cost efficacy of prophylaxis [26].

## Future Directions

The ideal management of VUR continues to evade us, despite all the progress made over the years, from the initial identification of the benefit of prophylaxis by Smellie et al. and the evolution of minimally invasive surgical modalities [27]. In this evolving management paradigm, the RiVUR study has potentially reaffirmed the benefit of prophylaxis, but has also allowed further analysis of the accrued data to evaluate additional factors that may positively or negatively impact VUR management. Since randomized controlled studies are difficult to perform in children, and can be expensive, every effort should continue to extract all of the findings from the data that have been collected from these large cohorts of patients.

## Conclusions

The RiVUR study was a robust and meticulously recorded trial, and several re-analyses have been undertaken that have provided multiple lessons for the management of VUR. The 50% reduction in the recurrence of UTI, lends credence to the role for the use of prophylaxis in children identified with VUR. The risk of long-term prophylaxis appears small, from a perspective of adverse events or development of antimicrobial resistance. The risk of recurrent infection is greater in children with concomitant BBD, and boys that were uncircumcised.

The radiologic grading of VUR can be controversial, even among well trained pediatric radiologists, which may impact treatment decisions and research endeavors. Although renal scarring was rare in the RiVUR cohort, secondary evaluation seems to suggest that prevention of recurrent infection does have an impact on the reduction of renal scarring.

Finally, although antibiotic prophylaxis appears to be marginally more expensive in children with VUR, this could be made much more cost effective, if the medication selected for the prophylactic regimen is even slightly less costly.

## Abbreviations:

RiVUR: Randomized intervention for Vesicoureteral Reflux
VUR: Vesicoureteral Reflux
UTI: Urinary Tract Infection
AAP: American Academy of Pediatrics
RBUS: Renal Bladder Ultrasound
VCUG: Voiding Cystourethrogram
TMP/SMZ: Trimethoprim/sulfamethoxazole
DMSA: Dimercaptosuccinic Acid
BBD: Bladder and Bowel Dysfunction
CUTIE: Careful Urinary Tract Evaluation Study
